# Orexin-A Excites Airway Vagal Preganglionic Neurons *via* Activation of Orexin Receptor Type 1 and Type 2 in Rats

**DOI:** 10.3389/fncel.2019.00478

**Published:** 2019-10-23

**Authors:** Yonghua Chen, Yuhong Guo, Xianxia Yan, Ming Zeng, Hong Chen, Dongying Qiu, Jijiang Wang

**Affiliations:** ^1^Department of Physiology and Pathophysiology, School of Basic Medical Sciences, Fudan University, Shanghai, China; ^2^Department of Neurobiology, School of Basic Medical Sciences, Fudan University, Shanghai, China; ^3^Department of Gerontology, Fudan University Affiliated Zhongshan Hospital, Shanghai, China

**Keywords:** orexin, preganglionic neuron, vagus nerve, synaptic transmission, airway resistance

## Abstract

Airway vagal nerves play a predominant role in the neural control of the airway, and augmented airway vagal activity is known to play important roles in the pathogenesis of some chronic inflammatory airway diseases. Several lines of evidence indicate that dysfunctional central orexinergic system is closely related to the severity of airway diseases, however, whether orexins affect airway vagal activity is unknown. This study investigates whether and how orexin-A regulates the activity of medullary airway vagal preganglionic neurons (AVPNs). The expression of orexin receptor type 1 (OX_1_R) and type 2 (OX_2_R) was examined using immunofluorescent staining. The effects of orexin-A on functionally identified inspiratory-activated AVPNs (IA-AVPNs), which are critical in the control of airway smooth muscle, were examined using patch-clamp in medullary slices of neonatal rats. Airway vagal response to injection of orexin-A into the magna cisterna was examined using plethysmography in juvenile rats. The results show that retrogradely labeled AVPNs were immunoreactive to anti-OX_1_R antibody and anti-OX_2_R antibody. Orexin-A dose-dependently depolarized IA-AVPNs and increased their firing rate. In synaptically isolated IA-AVPNs, the depolarization induced by orexin-A was blocked partially by OX_1_R antagonist SB-334867 or OX_2_R antagonist TCS OX2 29 alone, and completely by co-application of both antagonists. The orexin-A-induced depolarization was also mostly blocked by Na^+^/Ca^2+^ exchanger inhibitor KB-R7943. Orexin-A facilitated the glutamatergic, glycinergic and GABAergic inputs to IA-AVPNs, and the facilitation of each type of input was blocked partially by SB-334867 or TCS OX2 29 alone, and completely by co-application of both antagonists. Injection of orexin-A into the magna cisterna of juvenile rats significantly increased the inspiratory and expiratory resistance of the airway and consequently decreased the dynamic compliance of the lungs, all of which were prevented by atropine sulfate or bilateral vagotomy. These results demonstrate that orexin-A excites IA-AVPNs *via* activation of both OX_1_R and OX_2_R, and suggest that increased central synthesis/release of orexins might participate in the pathogenesis of airway diseases *via* over-activation of AVPNs.

## Introduction

The pulmonary branch of the parasympathetic (vagal) nervous system plays a key role in the neural control of airway function; and dysfunction of this vagal branch has long been suggested to participate in the pathogenesis of some chronic airway diseases such as bronchial asthma and obstructive sleep apnea syndrome (Lutz and Sułkowski, 2004; Lewis et al., 2006; Leung, 2009). The airway vagal tone is determined by central airway vagal preganglionic neurons (AVPNs), which project efferent fibers to postganglionic neurons innervating the smooth muscle, submucosal glands and vasculature of the airway (Baker et al., 1986; Undem et al., 1990; Dey et al., 1996; Maize et al., 1998; Hadziefendic and Haxhiu, 1999).

Using retrograde tracing techniques, previous studies have found that AVPNs are mainly located within three areas in the medulla: the compact portion of the nucleus ambiguus (cNA), the external portion of the NA (eNA) and the rostral portion of the dorsal motor nucleus of the vagus (DMV; Kalia and Mesulam, 1980; Haselton et al., 1992; Haxhiu et al., 1993; Haxhiu and Loewy, 1996; Kc et al., 2004). Functionally, the AVPNs within the DMV primarily innervate tracheobronchial secretory glands and vasculature, and activation of these neurons has little effect on airway resistance (Haselton et al., 1992; Kc et al., 2004). The AVPNs within the cNA send laryngeal nerve to control intrinsic laryngeal muscles (Irnaten et al., 2001a,b; Barazzoni et al., 2005; Okano et al., 2006; Chen et al., 2007). Only the AVPNs in the eNA are capable of altering the tension of airway smooth muscle upon activation (Iwase et al., 1992; Coon et al., 2000; Valic et al., 2001; Mueller et al., 2004). According to the distinct electrophysiological characteristics, AVPNs in the eNA are classified into inspiratory-activated (IA-) and inspiratory-inhibited (II-) AVPNs; and IA-AVPNs, which burst during the inspiratory phase, constitute the majority of AVPNs in the eNA (Chen et al., 2007, 2012; Qiu et al., 2011; Zhou et al., 2013). Consistently, a previous *in vivo* study in cats has found that some neurons in the para-tracheobronchial ganglion burst during the inspiratory phase and primarily project to the tracheobronchial smooth muscle, while others fire tonically during the expiratory phase and mostly project to the intercartilaginous spaces (Mitchell et al., 1987). It is reasonable to assume that the “bursting” postganglionic neurons are predominately controlled by IA-AVPNs while the “tonic” postganglionic neurons by II-AVPNs. Therefore, although different subpopulations of AVPNs may exert distinct but coordinated actions in controlling airway function, IA-AVPNs in the eNA may be critically important in controlling airway smooth muscle.

Orexins, including orexin-A and orexin-B (also known as hypocretin-1 and hypocretin-2), are a family of neuropeptides from the same precursor, which are exclusively produced by a subset of neurons in the lateral hypothalamus (de Lecea et al., 1998; Sakurai et al., 1998). Orexins play important roles in the neural control of a variety of physiological functions such as energy homeostasis, sleep-wake cycle, respiration, stress responses and visceral activities (Lubkin and Stricker-Krongrad, 1998; Sakurai et al., 1998; van den Pol et al., 1998; Chemelli et al., 1999; Young et al., 2005; Nakamura et al., 2007; Scammell and Winrow, 2011). Several lines of evidence indicate that the activity of AVPNs is modulated by orexins; and dysfunction of the central orexinergic system participates in the pathogenesis of some chronic airway diseases. Orexin-containing hypothalamic neurons project to the ventrolateral medulla of rats, and dense orexin-immunoreactive fibers and orexin receptor type 1 (OX_1_R) are found in the NA and nearby areas that roughly correspond to the location of AVPNs (Young et al., 2005). In a rat model of smoke-induced chronic obstructive pulmonary disease (COPD), the synthesis of orexin-A is increased in hypothalamic neurons; the content of orexin-A is increased in both the hypothalamus and medulla; and the expression of OX_1_R and orexin receptor type 2 (OX_2_R) in neurons of the ventrolateral medulla is up-regulated (Liu et al., 2010). Clinically, it has been indicated that plasma orexin-A level is closely associated with the severity of hypoxemia in COPD patients with hypercapnic respiratory failure (Zhu et al., 2011). However, it remains to be elucidated whether and how orexins modulate the activity of AVPNs, and as a result, alter the vagal control of airway function.

In the present study, the expression of OX_1_R and OX_2_R in retrogradely labeled AVPNs in the eNA was examined with immunofluorescent staining; the effect of orexin-A on the activity of IA-AVPNs in the eNA was examined in brainstem slices of neonatal rats with patch-clamp techniques; and the impact of orexin-A administrated into the cisterna magna on the inspiratory and expiratory resistance of the airway (R_i_ and R_e_), and consequently on the dynamic compliance of the lungs (C_dyn_), were evaluated with plethysmography in anesthetized juvenile rats. We aimed to test the hypothesis that orexins play an important role in the regulation of AVPNs.

## Materials and Methods

### Animals

All animal procedures were performed in compliance with the institutional guidelines at Fudan University (Shanghai, China), and in accordance with the National Institutes of Health guide for the care and use of laboratory animals. Immunofluorescent staining and electrophysiological experiments were performed in newborn (2- to 5-day-old) Sprague–Dawley (SD) rats of either sex; and *in vivo* experiments were performed in juvenile (2-week-old, 28–33 g body weight) male SD rats (Shanghai Slack Laboratory Animal Company Limited, Shanghai, China). A total of 152 rats were used. The authors have made maximal efforts to minimize the number and reduce the suffering of animals.

### Retrograde Fluorescent Labeling of AVPNs

AVPNs in the NA of newborn rats were retrogradely labeled as we have described previously (Chen et al., 2007). Briefly, a 2- to 3-day-old SD rat was anesthetized with inhalation agent halothane, and fixed on a plastic bag filled with ice-water mixture to lower body temperature and heart rate. The extra-thoracic trachea was exposed, and fluorochrome rhodamine (XRITC, Molecular Probes, USA; 1% solution, 0.2–0.5 μl) or fluorogold (Biotium company, Fremont, CA, USA; 4% solution, 0.2–0.5 μl) was injected into the trachea wall between the fourth and eighth tracheal cartilage *via* a glass pipette (tip diameter 30 μM) to retrogradely label AVPNs. The wound was closed and sutured with surgical silk (4.0). After surgery, the animal was put back in the same cage with the mother rat and the same litter, and allowed 48–72 h to recover.

### Immunohistochemical Experiments

Seven SD rats that had received injections of fluorogold in the trachea wall were anesthetized deeply with halothane and rapidly perfused transcardially with 0.9% buffered saline followed by 4% paraformaldehyde in 0.1 M PBS (pH 7.4). The perfused hind brains were removed from the animals and post-fixed with 4% paraformaldehyde in 0.1 M PBS (pH 7.4) for 24 h at 4°C. After cryoprotection by immersion in 30% sucrose in 0.1 M PBS at 4°C for 48 h, 30 μM-thick frozen sections of the medulla oblongata were prepared using a freezing microtome (Jung Histocut, Model 820-II, Leica, Germany) and stored at −20°C in a cryoprotectant solution.

After three washes in PBS, the free-floating sections were incubated for 45 min in PBS-Triton solution containing 5% normal donkey serum to block non-specific binding sites. Sections from four rats were incubated at 4°C overnight in a PBS-Triton solution containing rabbit anti-OX_1_R antibody (no. O4514, lot 017k1041, 1:200; Sigma, St. Louis, MO, USA). Sections from three rats were incubated at 4°C overnight in a PBS-Triton solution containing rabbit anti-OX_2_R antibody (no. AOR002, lot AN-01, 1:200; Alomone, Jerusalem, Israel). The sections were rinsed three times in PBS, and incubated with Texas Red-conjugated secondary antibodies (1:200; Santa Cruz Biotechnology, Santa Cruz, CA, USA) at room temperature for 1 h. The sections were washed and mounted on gelatin/alum-coated glass slides. A drop of Vecta Shield (Vector Laboratories, Burlingame, CA, USA) was applied to air-dried sections, and the slides were coverslipped. For each experiment, a series of parallel control experiments were performed to determine whether the primary or secondary antibody produced false-positive results. In these experiments, sections were stained with the primary and secondary antibodies in all possible combinations, in which a single immunoprobe was omitted. Omission of the primary or secondary antibody resulted in the absence of fluorescence in the slides, demonstrating that no false-positive results were obtained with these reagents.

Fluorescent signals were detected with a confocal laser scanning microscope (Olympus Fluoview FV1000, Olympus, Tokyo, Japan; or Zeiss LSM710, Carl Zeiss Inc., Oberkochen, Germany). Co-localization of fluorogold with OX_1_R or OX_2_R was identified by alternating between filters to view fluorogold and Texas red fluorescence, and by analyzing the merged images of the same individual sites.

### Brainstem Slice Preparation

The rat that had received rhodamine injection in the trachea wall was anesthetized deeply with halothane and decapitated at the supracollicular level. The hind brain was exposed and immersed in cold (4°C) artificial cerebral spinal fluid (ACSF) of the following composition (in mM): NaCl 124, KCl 3, KH_2_PO_4_ 1.2, CaCl_2_ 2.4, MgSO_4_ 1.3, NaHCO_3_ 26, D-glucose 10 and sucrose 10, and constantly bubbled with gas (95% O_2_, 5% CO_2_) at pH 7.4. The cerebellum was removed and the brainstem was isolated with the aid of a dissection microscope. The brainstem was secured in the slicing chamber of a vibratome (Leica VT 1000S, Leica Microsystems, Wetzlar, Germany) filled with the same ACSF. The rostral end of the brainstem was set upwards, and the dorsal surface was attached to an agar block facing the razor using superglue. The brainstem was sectioned serially at variable thickness in the transverse plane. Once the cNA was visible under the microscope, a single medullary slice of 700–900 μM thick, of which one to two hypoglossal rootlets in each lateral were retained, was taken for experiments. The thick medullary slice preparation, which contains the pre-Bötzinger complex, local circuits for motor output generation and hypoglossal motor nuclei, generates inspiratory-phase motor discharge in hypoglossal nerves (Smith et al., 1991).

### Electrophysiological Experiments

The slice was transferred into the recording chamber and submerged in flowing ACSF (8–11 ml/min flow rate). The rostral cutting plane of the slice was set upwards to allow fluorescent identification and patch-clamp recording of AVPNs in the eNA. The temperature was maintained at 23 ± 0.5°C, and the concentration of KCl in the ACSF was adjusted to 8–9 mM to allow steady recording of the inspiratory-like hypoglossal rhythm. Individual AVPNs in the eNA were identified by the presence of the fluorescent tracer using an Olympus upright microscope (Olympus American Inc., Center Valley, PA, USA) through a 40× water immersion objective. Voltage-clamp or current-clamp experiments were performed on the fluorescently identified individual AVPN. The patch pipette (2–4 MΩ) was advanced to touch the surface of the neuron and brief negative pressure was used to obtain a seal over 1 GΩ between the pipette tip and the cell membrane. Under cell-attached mode, the neurons that discharge rhythmically during the inspiratory phase are identified as IA-AVPNs. The membrane under the pipette tip was then ruptured with a pulse suction to gain whole-cell configuration. Neurons with a stable membrane potential that was more negative than −40 mV were accepted for further study. In voltage-clamp experiments, the neurons were normally clamped at −80 mV. To record glutamatergic postsynaptic currents, the patch pipettes were filled with a solution consisting of (in mM): K^+^ gluconate 150, MgSO_4_•7H_2_O 2, CaCl_2_ 0.1, HEPES 10, EGTA 1, K_2_ATP 2, Na_3_GTP 0.1, pH 7.3. With this pipette solution and holding voltage, the Cl^−^-mediated inhibitory synaptic currents were minimized and only excitatory synaptic events were detectable. IA-AVPNs display spontaneous excitatory postsynaptic currents (sEPSCs) during the inspiratory intervals and phase-locked inward currents during the inspiratory phase. To record the GABAergic or glycinergic postsynaptic currents, the patch pipettes were filled with a solution consisting of (in mM): KCl 150, MgCl_2_ 2, EGTA 2, HEPES 10, and Mg-ATP 2, pH 7.3. With this pipette solution, the Cl^−^-mediated currents induced by the activation of GABAergic and glycinergic receptors were recorded as inward currents. Only the K^+^ gluconate pipette solution was used when current-clamp experiments were performed. The osmolarity of the ACSF and the pipette solutions was adjusted to 320 mOsm/L before use.

The patch-clamp signal was amplified with an Axopatch 700B amplifier (sampling frequency, 10 kHz; filter frequency, 1 kHz) and digitized with a 1322A Digidata. The inspiratory-phase bursts of the hypoglossal rootlets were recorded using a suction electrode, amplified with a BMA-931 bioamplifier (5 kHz sampling frequency, 10–1,000 Hz bandpass, 50,000 times), electronically integrated (*τ* = 50 ms) with a MA-1000 Moving Averager (CWE Inc., Ardmore, PA, USA), and digitized with the 1322A Digidata. The digitized patch-clamp signal and hypoglossal activity were simultaneously fed into a computer and collected with the Clampex 9.2 software (Axon Instruments, Foster City, CA, USA).

### Plethysmographic Measurement of Airway Function

After initiation of anesthesia with pentobarbital sodium (70 mg/kg, i.p.), a male SD rat (2-week-old, 28–33 g) was fixed in the supine position, tracheotomized just below the larynx and cannulated. The rat was put in the prone position. The nape of neck was incised at the midline and subcutaneous fascia was cut. The atlanto-occipital membrane was exposed by blunt dissection of the neck muscles. The neck was gently bent forwards to distend the atlanto-occipital membrane. A PE-10 tube filled with ACSF was inserted into the cisterna magna through a hole punched in the middle of the ligament and fixed in place using silicone gel (Kwik-Cast; World Precision Instruments, Sarasota, FL, USA). The wound was closed and sutured with surgical silk (4.0). The rat was placed in the plethysmographic chamber of a lung-function analyzing system (AniRes2005, Beijing Bestlab High-Tech Company Limited, Beijing, China) with the tracheal cannula connected to a rodent ventilator outside the chamber through a hole on the chamber wall. The ventilator was set at a respiration rate of 90 breaths/min and a tidal volume of 0.2–0.28 ml, and the ratio of inspiration time to expiration time in a respiratory cycle was set at 1:2. The system automatically calculates and records R_i_, R_e_ and C_dyn_ simultaneously. In some experiments, 20 min prior to the injection of orexin-A into the magna cisterna, the vagus nerve was transected bilaterally at the thyroid cartilage level (bilateral vagotomy), or atropine sulfate was injected (0.5 mg/kg, i.p.) to block the vagal-mediated airway responses. Only male SD rats were used in these *in vivo* experiments to avoid any gender-related developmental change of the response to orexin-A.

### Drug Application

In electrophysiological experiments, the drugs were used globally in the bath. Orexin-A was applied for 4 min at the concentration indicated. Selective OX_1_R antagonist SB-334867 (10 μM), selective OX_2_R antagonist TCS OX2 29 (10 μM), or a mixture of both antagonists was used to block OX_1_R or/and OX_2_R as reported (Smart et al., 2001; Mould et al., 2014). KB-R7943 (30 μM) was used to inhibit the Na^+^/Ca^2+^ exchanger (NCX). These antagonists or inhibitor were applied at least 10 min prior to and throughout the subsequent use of orexin-A. Strychnine (1 μM) and picrotoxin (20 μM) were used to block glycinergic receptors and GABAergic receptors, respectively. 6-Cyano-7-nitroquinoxaline-2,3-dione (CNQX; 20 μM) and D-2-amino-5-phosphonovalerate (AP_5_; 50 μM) were used to block non-NMDA and NMDA glutamatergic receptors, respectively. To isolate the GABAergic spontaneous inhibitory postsynaptic currents (sIPSCs), CNQX, AP_5_ and strychnine were added in the bath. To isolate the glycinergic sIPSCs, CNQX, AP_5_ and picrotoxin were added in the bath. In some experiments, sodium channel blocker tetrodotoxin (TTX; 1 μM) was added in the bath to prevent action potential generation and polysynaptic action of drugs, and for recording of glutamatergic miniature excitatory postsynaptic currents (mEPSCs) or GABAergic or glycinergic miniature inhibitory postsynaptic currents (mIPSCs). In each slice, only one IA-AVPN was tested with drugs, and each drug was normally used only once. However, in some experiments in which SB-334867, TCS OX2 29 or KB-R7943 was used, orexin-A was applied for a second time after washout of these compounds to exclude the possible toxicity of antagonists or inhibitor. In *in vivo* experiments, 10 μl ACSF, with or without orexin-A (6 nmol), was injected into the magna cisterna. In each rat, orexin-A was injected only once.

### Data Analysis

The data and statistical analysis complied with the recommendations on experimental design and analysis in pharmacology (Curtis et al., 2015). All immunofluorescence images were color/contrast enhanced in ImageJ. Spontaneous and miniature synaptic currents were analyzed with the MiniAnalysis software (version 4.3.1; Synaptosoft Inc., Fort Lee, NJ, USA) with the minimal acceptable amplitude of 10 pA. The data from a 30 s to 1 min period of the maximal response after orexin-A application were analyzed, and compared with those from a similar period prior to drug application. When analyzing the sEPSCs during inspiratory intervals, the phase-locked inward currents during the inspiratory phase were ignored. The orexin-A-induced changes in the firing rate of IA-AVPNs, membrane potential, baseline current, and phase-locked inspiratory inward currents with respect to the duration, peak amplitude and area were analyzed with the Clampfit 9.2 software (Molecular Devices, LLC., Sunnyvale, CA, USA). For comparison of the dose-dependent effect of orexin-A on the firing rate of AVPNs during the inspiratory phases and during inspiratory intervals, data from five consecutive inspiratory cycles prior to drug application and during the maximal response period after drug application were analyzed. For comparison of the orexin-A-induced changes in membrane potential and baseline current, usually a 5-s data segment during control and that during the maximal response period were averaged. R_i_, R_e_ and C_dyn_ were analyzed with the software of Anires2005 lung-function analyzing system (Beijing Bestlab High-Tech Company Limited, Beijing, China). The values of R_i_, R_e_ and C_dyn_ in each rat were measured once from a 30-s period during control recording and during the maximal response period, and further averaged with the data obtained from other rats in the same group for comparison. When analyzing the firing rate during the inspiratory phase, sEPSCs, glycinergic sIPSCs, GABAergic sIPSCs, R_i_, R_e_ and C_dyn_, the values prior to orexin-A application, no matter whether single or multiple antagonists have been pre-applied, were set as the controls, and the data during orexin-A application were expressed as the fold of the control values (Curtis et al., 2015).

All statistical analyses were performed using the SPSS version 20 (IBM Corp., Armonk, NY, USA). Data are presented as mean ± SE. When two groups of data were compared, pair-sample student’s *t*-test was used. When more than two groups of data were compared, one-way ANOVA followed by Bonferroni or Dunnett correction (ANOVA-Bonferroni or ANOVA-Dunnett in brief) was used. Normality tests were run before parametric tests to make sure that the data follow a normal distribution. Significant differences were set at *P* < 0.05.

### Materials

SB-334867 and TCS OX2 29 were purchased from Tocris Bioscience (Bristol, UK); and rest of the drugs was purchased from Sigma-Aldrich (St. Louis, MO, USA).

## Results

### Retrogradely Labeled AVPNs in the eNA Showed Positive Immunoreactivity for Both OX_1_R and OX_2_R

**Figure 1:**
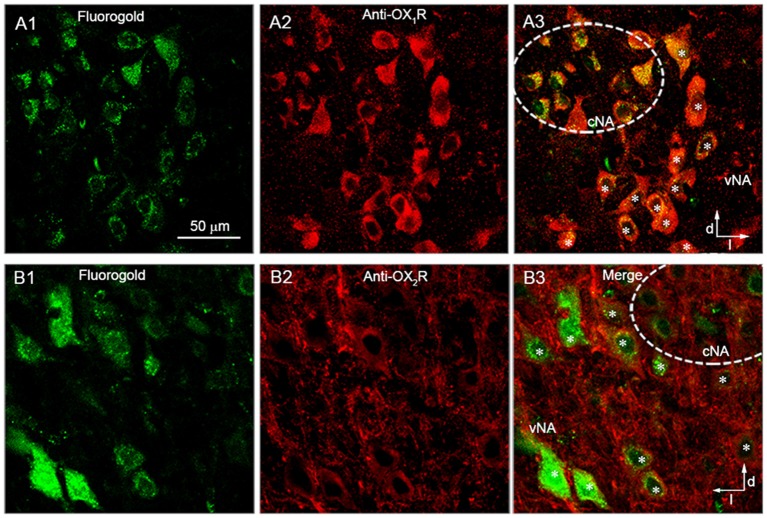
Retrogradely labeled airway vagal preganglionic neurons (AVPNs) in the external portion of the nucleus ambiguus (eNA) were positively immunoreactive to anti-orexin receptor type 1 (OX_1_R) and anti-OX_2_R antibodies.** (A1,B1)** Retrogradely labeled AVPNs (green) in the ventrolateral medulla. **(A2,B2)** Neurons in the ventrolateral medulla that were positively stained (red) by anti-OX_1_R **(A2)** and anti-OX_2_R antibody **(B2)**. **(A3,B3)** Merged images showing the positive immunoreactivity of retrogradely labeled neurons in the eNA to anti-OX_1_R (**A3**, marked by *) and anti-OX_2_R antibody (**B3**, marked by *). Note that retrogradely labeled AVPNs in the cNA were also positively immunoreactive to anti-OX_1_R antibody, but not anti-OX_2_R antibody. Dashed circles represent the range of cNA. Dorsal (d) and lateral (l) directions are indicated.

AVPNs retrogradely labeled by fluorogold were found in both the cNA and eNA (Figures 1A1,B1), as is consistent with the findings in our previous study (Chen et al., 2007). In eight brainstem slices from four rats, all (78/78) of the retrogradely labeled AVPNs identified in the eNA showed positive OX_1_R immunoreactivity (Figures 1A1–A3). In four slices from three rats, almost all (32/33) of the retrogradely labeled AVPNs identified in the eNA showed positive OX_2_R immunoreactivity (Figures 1B1–B3). In addition, while all of the retrogradely labeled AVPNs examined (>100) in the cNA showed positive OX_1_R immunoreactivity, none of them was positively immunoreactive for anti-OX_2_R antibody.

### Orexin-A Dose-Dependently Depolarized IA-AVPNs and Increased Their Firing Rate Both During the Inspiratory Phase and During Inspiratory Intervals

**Figure 2:**
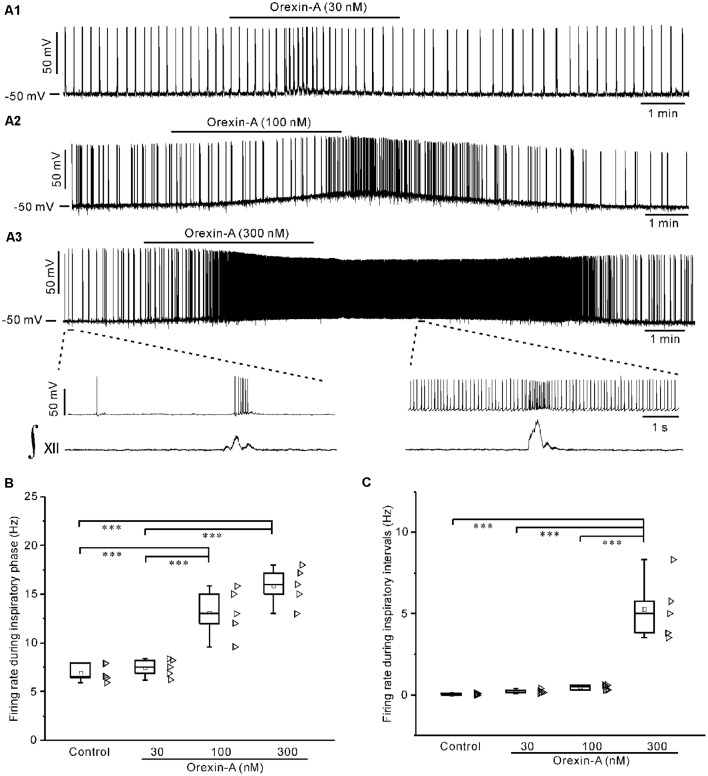
Orexin-A dose-dependently depolarized inspiratory-activated AVPNs (IA-AVPNs) and increased their firing rate during the inspiratory phase and inspiratory intervals. **(A1–A3)** Continuous current-clamp recordings in representative IA-AVPNs, showing the depolarization and increase of action potential discharge induced by orexin-A at 30 nM, 100 nM, and 300 nM, respectively. In panel **(A3)**, the segments indicated in top panel are shown in an enlarged time scale in middle panel, showing the orexin-A-induced increase of firing rate, along with the integrated simultaneous activity of hypoglossal rootlets (∫_XII_; bottom panel). **(B,C)** Box and scatter graphs showing the dose-dependent increase of firing rate induced by orexin-A during the inspiratory phase **(B)** and inspiratory intervals **(C)**, *n* = 5 for each box. ***P < 0.001, one-way ANOVA followed by Bonferroni test.

Under current-clamp mode, bath application of orexin-A (30 nM, 100 nM, 300 nM) dose-dependently depolarized IA-AVPNs, and increased the action potential discharge in all neurons tested. Orexin-A significantly increased the firing rate during the inspiratory phase at 100 nM and 300 nM (*P* < 0.001, *n* = 5 for both concentrations, ANOVA-Bonferroni), and significantly increased the firing rate during inspiratory intervals at 300 nM (*P* < 0.001, *n* = 5, ANOVA-Bonferroni). These responses started 1–2 min after application of 100 nM orexin-A and 30–60 s after application of 300 nM orexin-A, and usually disappeared 10 min after wash. The dose-dependent effect of orexin-A on the firing rate during the inspiratory phase and during inspiratory intervals was shown in Figures 2A1–A3, and summarized in Figures 2B,C. Because the effect of orexin-A at 100 nM is significant and modest, this concentration of orexin-A was then used in the following patch-clamp experiments.

### Orexin-A Depolarized Synaptically Isolated IA-AVPNs, Which Was Attenuated by SB-334867 or TCS OX2 29 Alone and Abolished by Co-application of Both Antagonists, and Was Mostly Antagonized by KB-R7943

**Figure 3:**
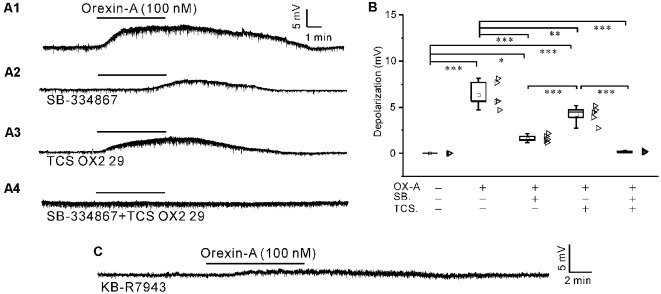
The depolarization induced by orexin-A in synaptically isolated IA-AVPNs was attenuated by pre-application of SB-334867 or TCS OX2 29 alone and abolished by co-application of both antagonists, and was mostly antagonized by KB-R7943.** (A1)** Continuous current-clamp recording showing the typical orexin-A-induced depolarization in an IA-AVPN, to which the neurotransmission was blocked by a cocktail of agents including TTX (1 μM), CNQX (20 μM), AP_5_ (50 μM), picrotoxin (20 μM) and strychnine (1 μM) in the perfusate. **(A2–A4)** The orexin-A-induced depolarization of IA-AVPNs in the presence of SB-334867 (10 μM; **A2)**, TCS OX2 29 (10 μM; **A3)** or both of them **(A4)** in addition to the cocktail. **(B)** Summarized data for the orexin-A-induced depolarization under different conditions (*n* = 5 for each box). OX-A, orexin-A; SB., SB-334867; TCS., TCS OX2 29. *, **, ***P < 0.05, 0.01, 0.001, respectively; one-way ANOVA followed by Bonferroni test. **(C)** Recording of a representative IA-AVPN in the presence of KB-R7943 (30 μM), showing that the orexin-A-induced depolarization was mostly antagonized.

After pretreatment of the slices with a cocktail of agents including TTX (1 μM), CNQX (20 μM), AP_5_ (50 μM), picrotoxin (20 μM) and strychnine (1 μM), orexin-A (100 nM) induced significant depolarization (*P* < 0.001 compared with the baseline value set as zero, *n* = 5, ANOVA-Bonferroni) in IA-AVPNs, as is shown by a representative experiment in Figure 3A1.

The orexin-A-induced depolarization was reduced by pre-application of SB-334867 (10 μM) or TCS OX2 29 (10 μM) alone and abolished by pre-application of both antagonists in combination. In the presence of SB-334867 (*n* = 5) or TCS OX2 29 (*n* = 5) alone, orexin-A still induced significant depolarization (*P* < 0.05 in the presence of SB-334867 and *P* < 0.001 in the presence of TCS OX2 29, respectively; ANOVA-Bonferroni), which is significantly smaller compared with that in the absence of any antagonist (*P* < 0.001 and *P* < 0.01, respectively; ANOVA-Bonferroni). Representative experiments are shown in Figures 3A2–A4; and summarized data are shown in Figure 3B. After 10 min washout of the antagonists (inhibitor), orexin-A (100 nM) still induced depolarization of similar amplitude in the same individual IA-AVPN (data not shown), which demonstrates that the diminishment of orexin-A-induced responses in the presence of these antagonists (inhibitor) was not due to general toxicity and/or fatigue of the preparation.

After pre-application of KB-R7943 (30 μM), a potent and selective inhibitor of the NCX, the orexin-A-induced depolarization was minimized (0.6 ± 0.0 mV, *n* = 5), which is not significantly different with the level before orexin-A application (set as zero; *P* > 0.05, pair-sample *t-test*). A representative experiment is shown in Figure 3C.

### Orexin-A Increased the Frequency and Amplitude of sEPSCs in IA-AVPNs, Which Was Attenuated by SB-334867 or TCS OX2 29 Alone, and Abolished by Co-application of Both Antagonists

**Figure 4:**
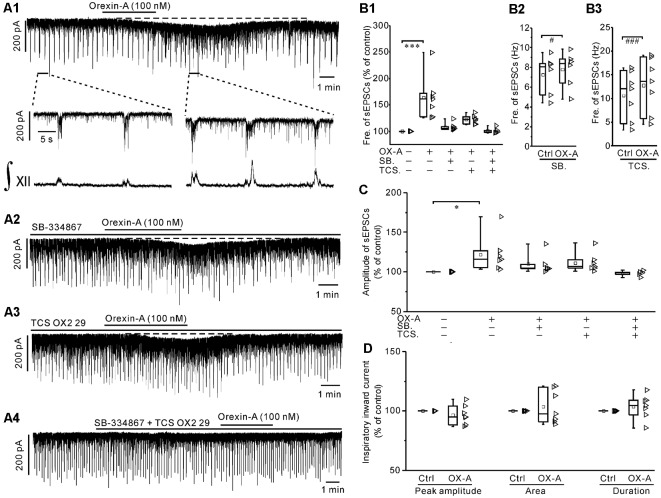
Orexin-A increased both the frequency and amplitude of spontaneous excitatory postsynaptic currents (sEPSCs) during inspiratory intervals in IA-AVPNs. **(A1)** Continuous voltage-clamp recording in a representative IA-AVPN, showing the increases in the frequency and amplitude of sEPSCs induced by orexin-A (100 nM), along with a slow inward current (top panel). The selected segments in top panel are shown in an enlarged time scale (middle panel), along with the integrated simultaneous activity of hypoglossal rootlets (∫_XII_; bottom panel). **(A2–A4)** Continuous voltage-clamp recordings in representative IA-AVPNs, showing the effect of orexin-A (100 nM) on the sEPSCs in the presence of SB-334867 (10 μM) or TCS OX2 29 (10 μM) alone, or both of them. Note that SB-334867 and TCS OX2 29 pre-applied in combination did not cause any change in the sEPSCs or phase-locked inspiratory inward currents of IA-AVPNs **(A4)**. **(B1,C)** Summarized data for the changes in the frequency **(B1)** and amplitude **(C)** of sEPSCs during application of orexin-A alone (*n* = 7) and in the presence of SB-334867 (*n* = 6), TCS OX2 29 (*n* = 7) or both (*n* = 5). *P < 0.05; ***P < 0.001; one-way ANOVA followed by Dunnett correction. **(B2,B3)** Summarized data for the frequency increase of sEPSCs during application of orexin-A in the presence of SB-334867 (B2, *n* = 6) or TCS OX2 29 (B3, *n* = 7). ^#^*P* < 0.05; ^###^*P* < 0.001; pair-sample *t-test*. **(D)** Summarized data for the peak amplitude, area and duration of phase-locked inspiratory inward currents of IA-AVPNs, showing the ineffectiveness of orexin-A. *n* = 7 for each box, pair-sample *t-test*. Fre., frequency; Ctrl, control; OX-A, orexin-A; SB., SB-334867; TCS., TCS OX2 29.

Orexin-A (100 nM) significantly increased the frequency of sEPSCs by 63.8 ± 15.3% (*P* < 0.001 vs. control, *n* = 7, ANOVA-Dunnett; Figures 4A1,B1) and significantly increased the amplitude by 22.1 ± 8.7% (*P* < 0.05 vs. control, *n* = 7, ANOVA-Dunnett; Figures 4A1,C). In consistent with the depolarization under current-clamp, orexin-A induced a slow inward current (Figure 4A1), of which the maximal value is 55.3 ± 4.7 pA (*n* = 7). In addition, orexin-A did not cause any significant change in the phase-locked inspiratory inward currents with respect to the peak amplitude, duration and area (*P* > 0.05 vs. controls, respectively, *n* = 7, pair-sample *t-test*; Figure 4D).

In the presence of SB-334867 (10 μM) or TCS OX2 29 (10 μM) alone, orexin-A did not cause any significant change of sEPSCs when the data are expressed in percentages of the controls and compared with ANOVA-Dunnett test (*P* > 0.05, *n* = 5 for both frequency and amplitude and for both antagonists; Figures 4A2,A3,B1,C). However, when the data are expressed as absolute values and compared with pair-sample *t-test*, orexin-A caused a slight but significant frequency increase of sEPSCs, both in the presence of SB-334867 alone (*P* < 0.05, *n* = 6, pair-sample *t-test*; Figures 4A2,B2) and in the presence of TCS OX2 29 alone (*P* < 0.001, *n* = 7, pair-sample *t-test*; Figures 4A3,B3).

After pretreatment with SB-334867 (10 μM) and TCS OX2 29 (10 μM) in combination, orexin-A (100 nM) no longer caused any significant change in the sEPSCs (*P* > 0.05, *n* = 5; ANOVA-Dunnett; Figures 4A4,B1,C). In addition, pre-application of SB-334867 or TCS OX2 29 each reduced, and co-application of them eliminated, the orexin-A-induced slow inward current (Figures 4A2–A4), as is consistent with the attenuation or abolishment of the orexin-A-induced depolarization by these antagonists. SB-334867 and TCS OX2 29, either pre-applied alone or in combination, did not cause any change in the sEPSCs of IA-AVPNs (Figures 4A2–A4). At the end of each experiment, bath application of CNQX (20 μM) and AP_5_ (50 μM) blocked all of the sEPSCs and inspiratory inward currents.

### Orexin-A Significantly Increased the GABAergic and Glycinergic sIPSCs of IA-AVPNs, All of Which Were Attenuated by SB-334867 or TCS OX2 29 Alone and Abolished by Co-application of Both Antagonists

**Figure 5:**
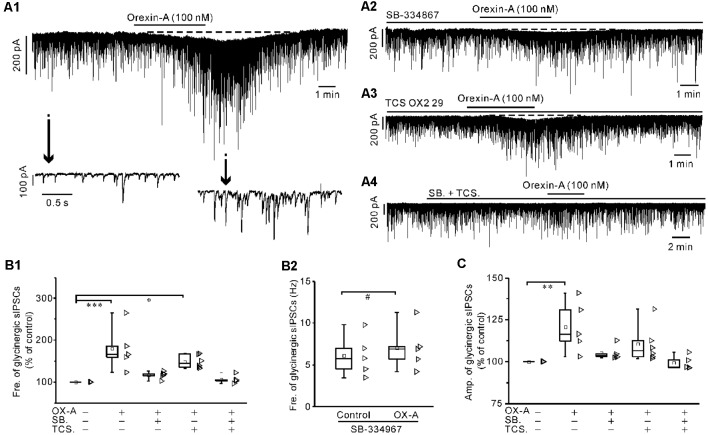
Orexin-A significantly increased the frequency and amplitude of the glycinergic spontaneous inhibitory postsynaptic currents (sIPSCs) of IA-AVPNs. **(A1)** Continuous voltage-clamp recording in a representative IA-AVPN, showing the orexin-A-induced increases in the frequency and amplitude of glycinergic sIPSCs, along with a slow inward current (upper panel). In the lower panel, recording segments selected from the upper panel are shown in an enlarged time scale. **(A2–A4)** Continuous voltage-clamp recordings in representative IA-AVPNs, showing the effect of orexin-A (100 nM) on the glycinergic sIPSCs in the presence of SB-334867 (10 μM), TCS OX2 29 (10 μM) or both. Note that SB-334867 and TCS OX2 29 pre-applied in combination did not cause any change in the glycinergic sIPSCs **(A4)**. **(B1,C)** Summarized data for the changes in the frequency** (B1)** and amplitude** (C)** of glycinergic sIPSCs during application of orexin-A alone (*n* = 5) and in the presence of SB-334867 (*n* = 5), TCS OX2 29 (*n* = 6) or both (*n* = 5). *, **, ***P < 0.05, 0.01, 0.001, respectively; one-way ANOVA followed by Dunnett correction. **(B2)** Summarized data for the frequency increase of the glycinergic sIPSCs induced by orexin-A in the presence of SB-334867 (*n* = 5). ^#^*P* < 0.05, paired-sample *t-test*. Fre., frequency; Amp., amplitude; OX-A, orexin-A; SB., SB-334867; TCS., TCS OX2 29.

**Figure 6:**
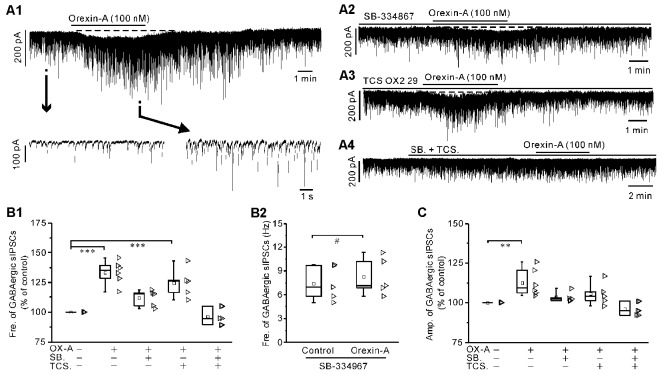
Orexin-A significantly increased the frequency and amplitude of the GABAergic sIPSCs in IA-AVPNs.** (A1)** Continuous voltage-clamp recording in a representative AVPN, showing the orexin-A-induced increases in the frequency and amplitude of GABAergic sIPSCs, along with a slow inward current (upper panel). In the lower panel, recording segments selected from the upper panel are shown in an enlarged time scale.** (A2–A4)** Continuous voltage-clamp recordings in representative IA-AVPNs, showing the effect of orexin-A (100 nM) on the GABAergic sIPSCs in the presence of SB-334867 (10 μM), TCS OX2 29 (10 μM) or both. Note that SB-334867 and TCS OX2 29 pre-applied in combination did not cause any change in the GABAergic sIPSCs **(A4)**. **(B1,C)** Summarized data for the changes in the frequency **(B1)** and amplitude** (C)** of GABAergic sIPSCs during application of orexin-A alone (*n* = 6) and in the presence of SB-334867 (*n* = 5), TCS OX2 29 (*n* = 5) or both (*n* = 6). **,***P < 0.01, 0.001, respectively; one-way ANOVA followed by Dunnett correction. **(B2)** Summarized data for the frequency increase of the GABAergic sIPSCs induced by orexin-A in the presence of SB-334867 (*n* = 5). ^#^*P* < 0.05, pair-sample *t-test*. Fre., frequency; Amp., amplitude; OX-A, orexin-A; SB., SB-334867; TCS., TCS OX2 29.

Orexin-A (100 nM) significantly increased the frequency of glycinergic sIPSCs by 79.9 ± 25.6% (*P* < 0.001 vs. control, *n* = 5, ANOVA-Dunnett) and increased the amplitude by 20.8 ± 7.1% (*P* < 0.01 vs. control, *n* = 5, ANOVA-Dunnett; Figures 5A1,B1,C). Orexin-A (100 nM) significantly increased the frequency of GABAergic sIPSCs by 33.5 ± 4% (*P* < 0.001 vs. control, *n* = 6, ANOVA-Dunnett) and increased the amplitude by 12.7 ± 3.7% (*P* < 0.01 vs. control, *n* = 6, ANOVA-Dunnett; Figures 6A1,B1,C).

In the presence of SB-334867 alone, orexin-A did not cause any significant change of either glycinergic or GABAergic sIPSCs when the data are expressed in percentages of the controls and compared with ANOVA-Dunnett test (for both types of sIPSCs, *P* > 0.05, *n* = 5; Figures 5A2,B1,C and Figures 6A2,B1,C). However, when the data are expressed as absolute values and compared with pair-sample *t-test*, orexin-A caused a slight but significant frequency increase of both glycinergic and GABAergic sIPSCs (For both types of sIPSCs, *P* < 0.05, *n* = 5; Figure 5B2 and Figure 6B2). In the presence of TCS OX2 29 alone, orexin-A caused a significant frequency increase of both glycinergic sIPSCs and GABAergic sIPSCs (*P* < 0.05, *n* = 5 for glycinergic sIPSCs; *P* < 0.001, *n* = 6 for GABAergic sIPSCs; ANOVA-Donnett), but did not cause a significant amplitude change of either types of sIPSCs (Figures 5A3,B1,C and Figures 6A3,B1,C). After pretreatment with SB-334867 (10 μM) and TCS OX2 29 (10 μM) in combination, orexin-A (100 nM) no longer caused any change in either glycinergic (*P* > 0.05, *n* = 5, ANOVA-Donnett) or GABAergic IPSCs (*P* > 0.05, *n* = 6, ANOVA-Donnett; Figures 5A4,B1,C and Figures 6A4,B,C). At the end of each experiment, bath application of strychnine (1 μM) or picrotoxin (20 μM) blocked the pharmacologically isolated glycinergic or GABAergic sIPSCs.

### Orexin-A Did Not Cause Significant Changes in the mEPSCs, Glycinergic mIPSCs or GABAergic mIPSCs of IA-AVPNs

**Figure 7:**
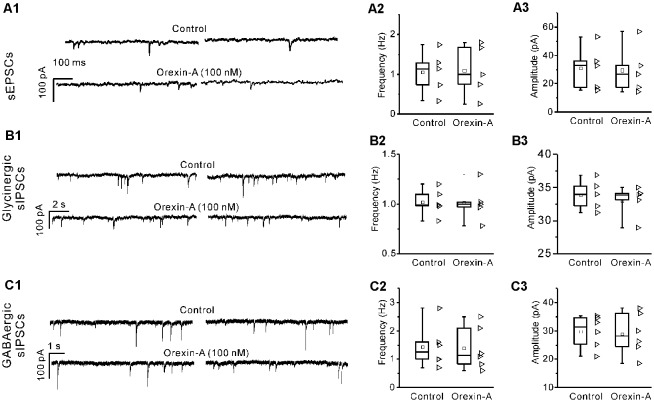
Orexin-A had no effect on the miniature excitatory postsynaptic currents (mEPSCs), glycinergic miniature inhibitory postsynaptic currents (mIPSCs) and GABAergic mIPSCs of IA-AVPNs. **(A1)** Representative voltage-clamp recording traces of the mEPSCs before and during application of orexin-A (100 nM), showing the ineffectiveness of orexin-A. Summarized data for the frequency and amplitude of mEPSCs are shown in** (A2,A3)**. Similar sets of data for glycinergic and GABAergic mIPSCs are shown in **(B1–B3)** and **(C1–C3)**. Fre, frequency; Amp, amplitude.

In the presence of TTX (1 μM), orexin-A (100 nM) did not cause any change in the mEPSCs (*P* > 0.05, *n* = 5, pair-sample *t-test*), glycinergic mIPSCs (*P* > 0.05, *n* = 5, pair-sample *t-test*) or GABAergic mIPSCs (*P* > 0.05, *n* = 6, pair-sample *t-test*) of IA-AVPNs. A representative experiment for mEPSCs is shown in Figure 7A1, and summarized data are shown in Figures 7A2,A3. Similar sets of data for glycinergic mIPSCs and GABAergic mIPSCs are shown in Figures 7B1–B3 and Figures 7C1–C3, respectively.

### Administration of Orexin-A Into the Cisterna Magna Increased R_i_ and R_e_ and Decreased C_dyn_ in Anesthetized Juvenile Rats

**Figure 8:**
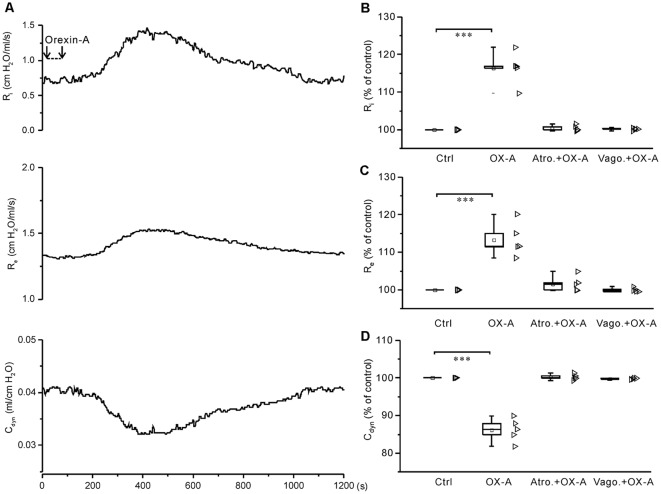
Application of orexin-A into the cisterna magna increased R_i_ and R_e_ and decreased C_dyn_, all of which were blocked by pretreatment with atropine or bilateral vagotomy. **(A)** Recording of R_i_, R_e_ and C_dyn_ in a representative rat, showing the changes induced by application of orexin-A (6 nmol in 10 μl ACSF) into the cisterna magna.** (B–D)** Summarized data for the changes of R_i_ **(B)**, R_e_ **(C)** and C_dyn_ **(D)** caused by orexin-A alone (*n* = 5) and after pretreatment with atropine (*n* = 5) or bilateral vagotomy (*n* = 5). ***P < 0.001; one-way ANOVA followed by Dunnett correction. Ctrl, control; OX-A, orexin-A; Atro., atropine; Vago., vagotomy.

In anesthetized juvenile rats, administration of ACSF (10 μl) into the cisterna magna caused little change in R_i_, R_e_ and C_dyn_. However, administration of orexin-A (6 nmol in 10 μl ACSF) into the cisterna magna significantly increased R_i_ and R_e_ by 16.4 ± 1.9% (*P* < 0.001, *n* = 5, ANOVA-Donnett) and 13.3 ± 2.0% (*P* < 0.001, *n* = 5, ANOVA-Donnett), respectively, and significantly decreased C_dyn_ by 13.8 ± 1.4% (*P* < 0.001, *n* = 5, ANOVA-Donnett). In contrast, in anesthetized juvenile rats pretreated with atropine sulfate (0.5 mg/kg, i.p.; *n* = 5) or bilateral vagotomy (*n* = 5), administration of orexin-A into the cisterna magna did not cause any significant change (Figure 8), suggesting that the responses induced by intracisternal orexin-A are mediated by airway vagal nerves.

## Discussion

This study for the first time supplies evidence that orexins are involved in the modulation of airway vagal activity. *In vivo*, intracisternal administration of orexin-A increased airway vagal activity, as were manifested by vagal-mediated increases in R_i_ and R_e_ and a decrease in C_dyn_. *In vitro*, orexin-A facilitated both the excitatory and inhibitory inputs of IA-AVPNs and caused depolarization *via* direct postsynaptic action, and consequently excited the IA-AVPNs.

The activity of AVPNs is largely dependent on their synaptic inputs, which include the excitatory glutamatergic and inhibitory GABAergic and glycinergic ones (Haxhiu et al., 2005). In this study, orexin-A caused significant frequency increases in the glutamatergic sEPSCs and GABAergic and glycinergic sIPSCs, but not in the glutamatergic mEPSCs and GABAergic and glycinergic mIPSCs. These results suggest that the action sites of orexin-A are most likely at the soma and/or dendrites of the neurons presynaptic to IA-AVPNs, while least likely at the terminals of them. Yet the possibility of polysynaptic actions of orexin-A cannot be ruled out. Moreover, orexin-A caused a significant amplitude increase in the glutamatergic sEPSCs and GABAergic and glycinergic sIPSCs, but not in the glutamatergic mEPSCs and GABAergic and glycinergic mIPSCs. These results suggest that the postsynaptic action of orexin-A does not alter the responses of IA-AVPNs to the glutamate, GABA or glycine released to them, and the orexin-A-induced amplitude increase is more likely due to the enhanced summation of the synaptic currents. This conclusion is further supported by the fact that so far the orexin-A-induced frequency increase of sEPSCs or sIPSCs was reduced to some extent, but not eliminated, by pre-application of OX_1_R antagonist or OX_2_R antagonist alone, the orexin-A-induced amplitude increase was no longer significant.

In addition to the presynaptic action, orexin-A also exert a direct postsynaptic action on IA-AVPNs, *via* activation of both OX_1_R and OX_2_R, as are demonstrated by the results that in synaptically isolated IA-AVPNs, the orexin-A-induced depolarization was partially blocked by SB-334867 or TCS OX2 29 alone and completely by these two antagonists in combination. This suggestion is further supported by the findings in our histochemical experiments that almost all of retrogradely labeled AVPNs in the eNA were stained by OX_1_R and OX_2_R antibodies, our results suggest that both OX_1_R and OX_2_R are involved in the postsynaptic action of orexin-A.

In contrast, although all of the retrogradely labeled AVPNs in the cNA were positively immunoreactive to OX_1_R antibody, none of them is positively immunoreactive to OX_2_R. Considering that AVPNs in the cNA retrogradely labeled from the tracheal wall are mostly laryngeal neurons (Chen et al., 2007), our results indicated that orexins might regulate laryngeal neurons and tracheobronchial-projecting AVPNs *via* activation of different subtype(s) of orexin receptors. This conclusion is consistent with the findings in previous studies, which found that OX_1_R and OX_2_R play different roles in the vagal control of visceral functions (Takahashi et al., 1999; Okumura et al., 2001; Kobashi et al., 2014). In particular, Kobashi et al. (2014) have demonstrated after administration of orexin into the fourth cerebral ventricle, activation of OX_1_R mediates the inhibition of reflex swallowing elicited by the superior laryngeal nerve in the rat.

The signaling pathway of orexin receptor activation is complex. In general, orexin receptors are G_q_- or G_i_/G_o_-protein coupled receptors. The membrane effects of orexin have been found to be mediated by several ionic mechanisms, which include inhibition of G-protein-regulated inward rectifying potassium (GIRK) channels and activation of voltage-gated calcium channels, transient receptor potential channels and NCX (van den Pol et al., 1998; Burdakov et al., 2003; Kohlmeier et al., 2004; Acuna-Goycolea and van den Pol, 2009; Peltonen et al., 2009). In this study, the orexin-A-induced depolarization in IA-AVPNs was mostly blocked by NCX inhibitor KB-R7943, suggesting that activation of the NCX is the key membrane ionic mechanism following activation of OX_1_R and/or OX_2_R in these neurons. However, we did notice that in the pre-existence of KB-R7943, orexin-A still induced slight but distinguishable depolarization in most of the neurons tested, indicating that the orexin-A-induced depolarization in IA-AVPNs might involve additional unknown membrane ionic mechanisms that might play minor roles.

The present study found that although orexin-A acts both pre- and post-synaptically on IA-AVPNs, its overall effect on these neurons is excitatory, as is manifested by prolonged membrane depolarization and increased firing rate not only during the inspiratory phases but also during inspiratory intervals. These results suggest that compared with the facilitation of excitatory inputs and the direct postsynaptic excitation, facilitated inhibitory inputs might only play a minor role in determining the excitability of these neurons. This conclusion is consistent with that of previous studies, which found that orexins are excitatory to the neurons of multiple brain regions associated with autonomic regulation such as the nucleus accumbens, locus coeruleus, tuberomammillary nucleus, nucleus tractus solitarius and amygdala (Eriksson et al., 2001; Hwang et al., 2001; Grabauskas and Moises, 2003; Mukai et al., 2009).

In conclusion, orexin-A increases the excitability of IA-AVPNs in the eNA through activation of both OX_1_R and OX_2_R, which presynaptically facilitates both the excitatory and inhibitory inputs and postsynaptically causes depolarization of IA-AVPNs *via* subsequent activation of NCX. These results implicate that dysfunctional central orexinergic system might contribute to the pathogenesis of some airway diseases *via* altered modulation of AVPNs.

## Data Availability Statement

The datasets generated for this study are available on request to the corresponding author.

## Ethics Statement

The animal study was reviewed and approved by Laboratory Animal Ethics Committee, School of Basic Medical Sciences, Fudan University.

## Author Contributions

YC and JW designed the study and prepared the manuscript. YC performed the majority of the experiments. YG, XY, MZ, HC, and DQ performed the minority of the experiments and helped in revision of the manuscript.

## Conflict of Interest

The authors declare that the research was conducted in the absence of any commercial or financial relationships that could be construed as a potential conflict of interest.

## Abbreviations

ACSF: artificial cerebral spinal fluid
AP_5_: D-2-amino-5-phosphonovalerate
AVPNs: airway vagal preganglionic neurons
C_dyn_: dynamic compliance of the lungs
cNA: compact portion of the nucleus ambiguus
CNQX: 6-Cyano-7-nitroquinoxaline-2,3-dione
COPD: chronic obstructive pulmonary disease
DMV: dorsal motor nucleus of the vagus
eNA: external portion of the nucleus ambiguus
IA-AVPNs: inspiratory-activated AVPNs
II-AVPNs: inspiratory-inhibited AVPNs
mEPSCs: miniature excitatory postsynaptic currents
mIPSCs: miniature inhibitory postsynaptic currents
NCX: Na^+^/Ca^2+^ exchanger
OX_1_R: orexin receptor type 1
OX_2_R: orexin receptor type 2
R_e_: expiratory resistance of the airway
R_i_: inspiratory resistance of the airway
sEPSCs: spontaneous excitatory postsynaptic currents
sIPSCs: spontaneous inhibitory postsynaptic currents
TTX: tetrodotoxin.
